# Targeting the polyadenylation factor EhCFIm25 with RNA aptamers controls survival in *Entamoeba histolytica*

**DOI:** 10.1038/s41598-018-23997-w

**Published:** 2018-04-09

**Authors:** Juan David Ospina-Villa, Alexandre Dufour, Christian Weber, Esther Ramirez-Moreno, Absalom Zamorano-Carrillo, Nancy Guillen, César Lopez-Camarillo, Laurence A. Marchat

**Affiliations:** 1 Instituto Politécnico Nacional, Escuela Nacional de Medicina y Homeopatía, Guillermo Massieu Helguera 239, Fracc. La Escalera Ticoman, CP 07320 Ciudad de México, Mexico; 2 Institut Pasteur, Unité d’Analyse d’Images Biologiques, 25 Rue du Dr Roux, F-75015 Paris, France; 3 Centre National de la Recherche Scientifique CNRS UMR 3691, 25 Rue du Dr Roux, F-75015 Paris, France; 4 Institut Pasteur, Unité d’Imagerie et Modélisation, 28 rue du Docteur Roux, 75015 Paris, France; 5 Centre National de la Recherche Scientifique, CNRS-ERL9195, 25 Rue du Dr Roux, F-75015 Paris, France; 6 Universidad Autónoma de la Ciudad de México, Posgrado en Ciencias Genómicas, San Lorenzo 290, Col. Del Valle, CP 03100 Ciudad de México, Mexico

## Abstract

Messenger RNA 3′-end polyadenylation is an important regulator of gene expression in eukaryotic cells. In our search for new ways of treating parasitic infectious diseases, we looked at whether or not alterations in polyadenylation might control the survival of *Entamoeba histolytica* (the agent of amoebiasis in humans). We used molecular biology and computational tools to characterize the mRNA cleavage factor EhCFIm25, which is essential for polyadenylation in *E*. *histolytica*. By using a strategy based on the systematic evolution of ligands by exponential enrichment, we identified single-stranded RNA aptamers that target EhCFIm25. The results of RNA-protein binding assays showed that EhCFIm25 binds to the GUUG motif *in vitro*, which differs from the UGUA motif bound by the homologous human protein. Accordingly, docking experiments and molecular dynamic simulations confirmed that interaction with GUUG stabilizes EhCFIm25. Incubating *E*. *histolytica* trophozoites with selected aptamers inhibited parasite proliferation and rapidly led to cell death. Overall, our data indicate that targeting EhCFIm25 is an effective way of limiting the growth of *E*. *histolytica in vitro*. The present study is the first to have highlighted the potential value of RNA aptamers for controlling this human pathogen.

## Introduction

The processing of pre-messenger RNA (pre-mRNA) at the 3′-untranslated region (3′-UTR) is an essential maturation step; it increases mRNA stability, facilitates export of pre-mRNA from the nucleus to the cytoplasm, and enhances mRNA translation efficiency. The key steps in this essential event in the life of an RNA consist of pre-mRNA 3′-end cleavage and then polyadenylation. These reactions are determined by a multistep mechanism in which specific RNA sequence motifs are recognized by multiprotein complexes, including cleavage and polyadenylation specificity factor (CPSF), cleavage stimulating factor (CstF), and cleavage factors Im and IIm (CFIm and CFIIm)^1–4^. CPSF, CstF and CF subunits are conserved among all eukaryotic organisms, including several unicellular parasites^5,6^. We focused on *Entamoeba histolytica*. This parasite is the causative agent of human amoebiasis, an infectious disease that still represents a major health problem in many developing countries worldwide. The symptoms of human amoebiasis range from asymptomatic infection, diarrhoea, dysentery, fulminant colitis and peritonitis to the development of potentially lethal extraintestinal abscesses (mainly in the liver)^7,8^. Moreover, the severe adverse drug reactions associated with currently available pharmacological treatments (metronidazole and other nitroimidazole compounds) and the decrease in *E*. *histolytica* drug susceptibility have created an urgent need for alternative, novel, specific treatments^9,10^.

The pathogenicity of *E*. *histolytica* has been linked to both host factors and genomic and transcriptomic factors in the parasite^11,12^. However, little is currently known about how gene expression is regulated in this pathogen. Nevertheless, DNA/RNA motifs and nuclear factors involved in transcription, splicing, and mRNA 3′ end processing have been described^5,13,14^. In particular, protein amino-acid (aa) sequence comparisons suggest that the amoeba’s pre-mRNA 3′-end processing machinery is in an intermediate evolutionary position between mammals and yeast. Furthermore, the presence of non-canonical poly(A) polymerases adds complexity to the mRNA 3′ end formation process in this single-celled eukaryote^5^. *E*. *histolytica* only has the 25 kDa subunit of CFIm (EhCFIm25)^5^, whereas active CFIm in humans is a heterotetramer complex comprising two 25 kDa subunits that interact with a dimer of 59 or 68 kDa subunits^15–17^. CFIm25 belongs to the Nudix hydrolase superfamily, and is an essential regulator of poly(A) site selection, and polyadenylation/cleavage reactions in eukaryotic cells^18–20^. The absence of higher molecular mass subunits in *E*. *histolytica*’s CFIm suggests that (i) mRNA polyadenylation has a particular mechanism in the parasite, and (ii) EhCFIm25 has central role^5^. Although EhCFIm25 conserves the characteristic features of its human orthologue, it possesses several important differences. Notably, three of the four glutamate residues at positions 154, 157 and 158 in the conserved Nudix box are replaced by lysine, and the last glycine residue of the motif is replaced by the hydrophilic residue serine. EhCFIm25 interacts with the poly(A) polymerase EhPAP^21^ and the transcriptional coactivator EhPC4 (our unpublished data), which is related to virulence, DNA replication and multinucleation in *E*. *histolytica*^22,23^. EhCFIm25 also interacts with mRNA 3′UTR through the conserved Leu135 and Tyr236 residues^24^, although the RNA binding motif has yet to be identified.

It has been demonstrated that several components of the polyadenylation machinery may be valuable therapeutic targets in protozoan parasites (namely CPSF-30 (CPSF4) in *Trypanosoma brucei* and CPSF-73 (CPSF3) in *Toxoplama gondii* and *Plasmodium* *falciparum*)^25–28^. Furthermore, we recently observed that EhCFIm25 silencing induces cell death and decreases virulence capacity, prompting us to hypothesize that EhCFIm25 may be a relevant target for *E*. *histolytica* control^29^. To test this hypothesis, we applied two powerful molecular biology approaches: (i) the development of aptamers that bind to EhCFIm25 and (ii) delivery aptamers to trophozoites via soaking. Aptamers are single-stranded (ss) RNA or DNA oligonucleotides whose unique three-dimensional structure enables them to interact with a specific target molecule (*i*.*e*. EhCFIm25 in the present case). A large body of biomedical research has shown that aptamers are better than other tools (e.g. antibodies) at detecting and inhibiting target molecules in diagnostics, therapeutics, and drug development^30^. In the present work, we used a systematic evolution of ligands by exponential enrichment (SELEX) protocol to identify RNA aptamers that target the EhCFIm25 protein. Thanks to RNA-protein binding assays and molecular modelling, we discovered that EhCFIm25 bound to the aptamers’ GUUG motif *in vitro*. Moreover, we demonstrated that ingestion of these aptamers dramatically inhibited *E*. *histolytica*’s growth.

## Methods

### Cell cultures

*E*. *histolytica* trophozoites (HMI:IMSS) were grown at 37 °C in TYI-S-33 medium with 20% bovine serum, 100 U/ml penicillin and 100 μg/ml streptomycin^31^. RNAse III-deficient *Escherichia coli* strain HT115 (rnc14::DTn10) was grown at 37 °C in LB broth for plasmid construction or 2YT broth for double-stranded (ds) RNA expression, in the presence of ampicillin (100 mg/ml) and tetracycline (10 mg/ml)^32^.

### Expression and purification of the recombinant EhCFIm25 protein

Competent *E*. *coli* BL21 (DE3) pLysS bacteria were transformed with the pRSET-*EhCFIm25* plasmid^21^. The EhCFIm25 was expressed with 1 mM isopropyl beta-D-thiogalactopyranoside (IPTG) and purified by Ni^2+^-NTA affinity chromatography (QIAGEN). The identity and integrity of the histidine-tagged EhCFIm25 protein was confirmed by 10% SDS-PAGE and Western blot assays using anti-6×-His tag antibodies (Roche) at 1:10000 dilution and the ECL Plus Western blotting detection system (Amersham).

### SELEX strategy

**Figure 1:**
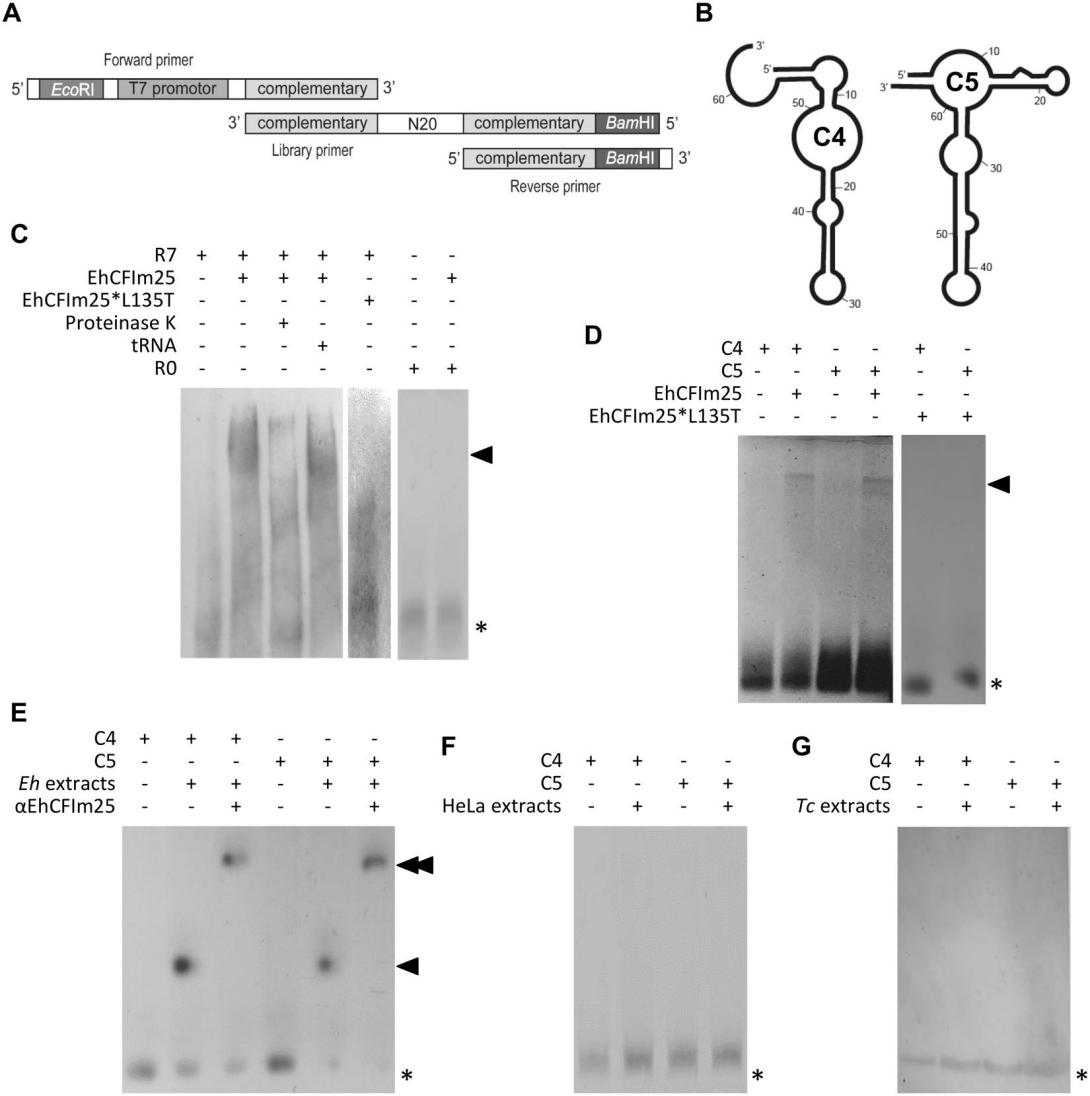
Identification of aptamers against EhCFIm25. (**A**) Schematic representation of ssDNA oligonucleotides used to generate the ssRNA library for the SELEX protocol. (**B**) The predicted secondary structures of the C4 and C5 aptamers. (**C**–**G**) The RNA-electrophoretic mobility shift assay (REMSA). Proteins were incubated with a biotin-labelled RNA probe, and the RNA-protein complexes were resolved via PAGE and chemiluminescence assays. (**C**) A REMSA of the R7 aptamer population with wild-type EhCFIm25 and mutant EhCFIm25*L135T proteins. Proteinase K (1 µg), unspecific competitor (tRNA) and RNA molecules from the first round of SELEX (R0) were used as controls. The image results from the grouping of gels cropped from different parts of the same gel and a different gel. (**D**) A REMSA of C4 and C5 aptamers with wild-type EhCFIm25 and mutant EhCFIm25*L135T proteins. The image results from the grouping of gels cropped from two different gels. (**E**) A REMSA of C4 and C5 aptamers with *E*. *histolytica* protein extracts. Anti-EhCFIm25 antibodies were used as controls. (**F** and **G**) A REMSA of C4 and C5 aptamers with protein extracts from HeLa cells (**F**) and *Trypanosoma cruzi* parasites (**G**). *Eh: E*. *histolytica*; *Tc: T*. *cruzi*; single arrowhead: the RNA-protein complex; double arrowhead: the RNA-protein-antibody complex; asterisk: the free probe.

RNA aptamers targeting EhCFIm25 were obtained using the SELEX protocol with some modifications^33^. First, we designed library primers with a region of 20 random nucleotides flanked by two conserved sequences (5′-TTACAGCAACCACCGGGGATCCATGGGCACTATTTATATCAAC(N)_20_AATGTCGTTGGTGGCCC-3′), a forward primer with a *Eco*RI site, the T7 promotor region and a complementary region to the conserved 3′ end of library primers, and a reverse primer with a *Bam*HI site and a complementary region to the conserved 5′ end of library primers (Fig. 1A). Then, library and forward primers (100 µM) were mixed with 10 mM dNTPs and 5 U Klenow enzyme (NEB) in Klenow buffer, for 1 h at 37 °C to obtain dsDNA. The ssRNA fragments potentially corresponding to about 4^20^ sequences were *in vitro* transcribed using 1 U T7 RNA polymerase (NEB), purified with PCA (Phenol:Chloroform:Isoamyl Alcohol 25:24:1) and quantified. Finally, they were passed throughout a Ni^2+^-NTA column previously coated with the histidine-tagged EhCFIm25 protein. After washing, bound aptamers were eluted with NT2 buffer, purified with PCA and incubated with the reverse primer and the M-MLV Reverse Transcriptase enzyme (Thermo Fisher SCIENTIFIC). The resulting cDNA fragments were PCR-amplified using forward and reverse primers with the Platinum Taq DNA Polymerase High Fidelity (Thermo Fisher SCIENTIFIC) as follows: 94 °C for 3 min; 30 cycles at 94 °C for 1 min, 40 °C for 1 min, 72 °C for 1 min; plus a final extension step at 72 °C for 20 min. Then, ssRNA were *in vitro* transcribed as described above to perform a new round of selection (R) (Supplementary Figure S1). Seven rounds of selection (R7) were performed to select aptamers with affinity for the EhCFIm25 protein. Finally, PCR products were cloned into the *Eco*RI and *Bam*HI sites of the pRSET A plasmid and sequenced using an Applied Biosystems 3500 Genetic Analyzer in the Unit of Molecular Biology at UNAM-Mexico.

### RNA-Electrophoretic Mobility Shift Assays (REMSA)

Selected aptamers (C4 and C5), and the 3′UTR of *thioredoxin* (EHI_021560), and *60SRibL7* (EHI_025830) genes of *E*. *histolytica*, were *in vitro* transcribed from pGEMT-*Ehthio*, pGEM-*Ehrib* plasmids (GenScript), pRSET-*C4* and pRSET-*C5* plasmids, respectively, using the MEGAshortscript T7 Kit (Ambion). These molecules and RNA molecules selected from the seventh round of the SELEX protocol (R7), were labeled with the Biotin RNA Labeling Mix (Roche). Their size and integrity were verified by agarose gel electrophoresis and chemiluminescence (Chemiluminescent Nucleic Acid Detection Module, Pierce). Then, RNA probes (100 ng/µl) were mixed with EhCFIm25 (20 µg) or *E*. *histolytica* extracts (50 µg) at room temperature for 20 min; RNA-protein complexes were resolved at 100 V for 1 h on pre-electrophoresed 10% non-denaturing PAGE and detected using LightShift™ Chemiluminescent RNA EMSA Kit (Thermo Fisher SCIENTIFIC). In some assays, proteinase K (20 U), tRNA (0.1 mg/mL), RNA fragments of the first round of SELEX (R0), the mutant protein (EhCFIm25*L135T)^24^ and protein extracts from HeLa cells or *Trypanosoma cruzi* parasites were used as controls.

### Modeling and docking experiments

The secondary structure of C4 and C5 aptamers was predicted using the Unified Nucleic Acid Folding and hybridization package (http://unafold.rna.albany.edu/)^34,35^. The three-dimensional structure of EhCFIm25 (C4M2T1, 255 residues) was predicted by homology modeling with the MODELLER package^36^ (http://www.unamur.be/sciences/biologie/urbm/bioinfo/esypred/) using as template the crystal structure of the human CFIm25 protein (chain A) in complex with the UUGUAU RNA molecule (chain C) (PDB 3MDG), and validated with the Verify_3D software^37^ (http://services.mbi.ucla.edu/Verify_3D/). On the other hand, we used the RNAComposer online software (http://rnacomposer.cs.put.poznan.pl/) to obtain a PDB structure of the GUUG RNA motif. Then, both molecules were individually submitted to molecular dynamics (MD) simulations as described below to obtain the most relaxed structures. Finally, we used the NPDock (Nucleic acid-Protein Dock) web server (http://genesilico.pl/NPDock/) for modeling the GUUG-EhCFIm25 complex structure using default parameters^38^. The best scoring model was chosen to analyze contacts between EhCFIm25 residues and the GUUG molecule by the CMA (Contact Map Analysis) software (http://ligin.weizmann.ac.il/cma/). For some analyses, we used the Swiss-PdbViewer 4.10 software (http://www.expasy.org/spdbv/)^39^ to obtain the mutant EhCFIm25*L135T protein.

### Molecular dynamics simulation

MD simulations of EhCFIm25 protein alone or interacting with the GUUG motif were performed using the GROMACS 5.1 package and the CHARMM27 v2.0 force field for proteins^40,41^. Molecules were centered in a cubic box at 1.0 nm from edges, using periodic boundary conditions and three points equilibrated solvent model. Cl^−^ or Na^+^ ions were added to neutralize the system that was equilibrated under a canonical or NVT ensemble to stabilize the temperature, and then under a NPT ensemble to equilibrate the pressure using the Parrinello-Rahman barostat^42^. Finally, we performed the position restraints and the MD production at 300 °K running a first step of 1 ns simulation followed by an extension step of 50 ns.

### Analysis of MD trajectories

First, we used the Trjconv tool of GROMACS to correct any periodicity in the system. After that, the atomic characteristics of EhCFIm25 protein alone or with the GUUG motif were compared using the analysis tools included in GROMACS software. RMSD (root mean square deviation) and RMSF (root mean square fluctuations) values of Cα backbone were calculated. The electrostatic potential was determined using the PDB2PQR server and the APBS software package^43^ (http://nbcr-222.ucsd.edu/pdb2pqr_2.1.1/). The simulation trajectory was visualized in the VMD 1.9.3 *beta1* software^44^ (http://www.ks.uiuc.edu/Research/vmd/).

### Blocking of EhCFIm25 by C4 and C5 aptamers

To deliver aptamers into *E*. *histolytica* trophozoites, we used bacterially expressed dsRNA and parasite-soaking experiments as described^45^. Briefly, DNA sequences corresponding to C4 and C5 aptamers were PCR amplified from pRSET-*C4* and pRSET-*C5* plasmids, respectively, and cloned into the *Sma*I and *Xho*I sites of the pL4440 vector. DNA sequencing was performed to verify the resulting pL4440-*C4* and pL4440-*C5* plasmids. Then, competent *E*. *coli* HT115 cells were independently transformed with each plasmid and dsRNA synthesis was induced with 2 mM IPTG for 4 h at 37 °C. Bacterial pellet was mixed with 1 M ammonium acetate and 10 mM EDTA, incubated with phenol∶chloroform∶isoamyl alcohol (25∶24∶1) and centrifuged. Nucleic acids were washed with isopropanol and 70% ethanol. DNase I (Invitrogen) and RNase A (Ambion) were added to eliminate ssRNA and dsDNA molecules, *C4*-dsRNA and *C5*-dsRNA were washed with isopropanol and 70% ethanol, analyzed by agarose gel electrophoresis and quantified. Finally, purified dsRNA molecules (100 μg/ml) were added to trophozoites cultures (5.0 × 10^4^) at 37 °C. Parasites grown in standard conditions or incubated with the *gfp-*dsRNA (green-fluorescent protein) were used as controls.

### Cell proliferation and viability assays

Each day, *E*. *histolytica* trophozoites were counted in a Neubauer chamber to evaluate cell proliferation. Simultaneously, living trophozoites were identified from the Trypan blue dye exclusion test. Experiments were performed twice in duplicate.

### dsRNAs stability

Purified *C4*-dsRNA or *C5*-dsRNA were mixed with complete TYI-S medium (100 μg/ml) at 37 °C. Each day, dsRNA molecules were resolved through 1% agarose gel electrophoresis and GelRed staining to evaluate their integrity.

## Results

### Identification and purification of RNA aptamers targeting EhCFIm25

Aptamers have been used to identify the RNA binding site in human CFIm25, and they inhibited the protein’s functions^19^. We identified RNA aptamers against the EhCFIm25 protein by using a SELEX affinity strategy with the recombinant His-tagged protein and a random ssRNA library (Fig. 1A). The canonical steps of the SELEX process were incubation, separation, elution, amplification, and purification of the single-stranded oligonucleotides; this process was repeated for several rounds (Supplementary Figure S1). At each round of selection in the SELEX process, PCRs confirmed the recovery of aptamers (data not shown). The pool of aptamers obtained from round seven (R7) was biotin-labelled, and its ability to bind to EhCFIm25 was confirmed in an RNA-electrophoretic mobility shift assay (REMSA). The resulting RNA-protein complex disappeared when proteinase K was added but was maintained in the presence of tRNA (used as a nonspecific competitor). In contrast, an RNA-protein complex was not observed when we used the mutant EhCFIm25*L135T protein, in which the Leu135 residue had been replaced by Thr. And this is not because this mutant polypeptide lacks RNA binding affinity since it is still able to the RNA 3′end of the *EhPgp5* gene^24^. Similarly, EhCFIm25 did not form a complex with RNA fragments from SELEX R0 (Fig. 1C).

### *In vitro* verification of isolated RNA aptamers

Two aptamers (referred to as C4 and C5) from the last round of selection (R7) were chosen for further characterization because of their high expected specificity and affinity for the EhCFIm25 protein (Fig. 1B; Supplementary Figure S2). The aptamers were transcribed and biotin-labelled for the REMSA. Both C4 and C5 interacted with the recombinant EhCFIm25 protein but not with the mutant EhCFIm25*L135T (Fig. 1D). Moreover, C4 and C5 were able to form a complex with total protein extract from *E*. *histolytica*. The addition of anti-EhCFIm25 antibodies produced a supershift in the RNA-protein complex, indicating that C4 and C5 also bind to the endogenous EhCFIm25 protein (Fig. 1E). However, neither C4 nor C5 were able to bind to CFIm25 proteins from HeLa cells (UniProtKB/Swiss-Prot: O43809) or *T*. *cruzi* parasites (GenBank: EKG06513.1) used as controls, which share homology (32% and 30%, respectively) with EhCFIm25 (Fig. 1F and G).

### EhCFIm25 protein binds to the GUUG motif *in vitro*

**Figure 2:**
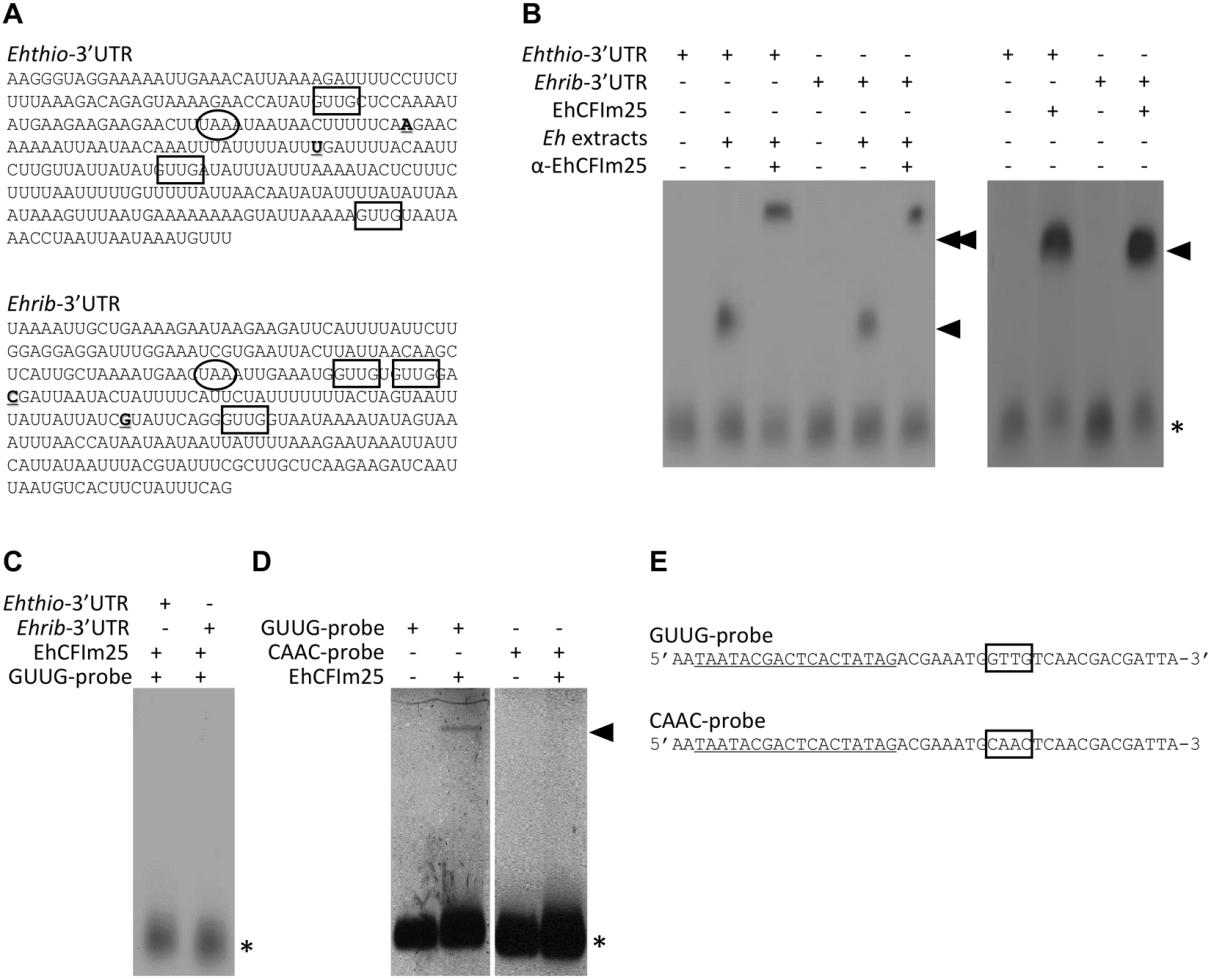
EhCFIm25 binds to the GUUG motif *in vitro*. (**A**) Sequence of the *Ehthio*- and *Ehrib*-3′UTR probes used in REMSAs. Box: the GUUG motif; circle: the stop codon: bold and underlined nucleotides: the cleavage and polyadenylation site. (**B**–**D**) The REMSA results. Proteins were incubated with a biotin-labelled RNA probe, and RNA-protein complexes were resolved via PAGE and chemiluminescence assays. (**B**) A REMSA of *Ehthio-* and *Ehrib*-3′UTR with *E*. *histolytica* extracts and the recombinant EhCFIm25 protein. Anti-EhCFIm25 antibodies were used as a control. (**C**) A REMSA of *Ehthio*- and *Ehrib*-3′UTR with the recombinant EhCFIm25 protein. The GUUG-containing oligonucleotide (GUUG-probe) was used as a competitor. (**D**) A REMSA of the GUUG-probe with recombinant EhCFIm25. The CAAC-containing oligonucleotide (CAAC-probe) was used as control. *Eh: E*. *histolytica*; single arrowhead: the RNA-protein complex; double arrowhead: the RNA-protein-antibody complex; asterisk: the free probe. (**E**) Sequence of the GUUG- and CAAC-probes. Box: the GUUG motif; the T7 promoter sequence is underlined.

A set of 12 aptamers from R5, R6 and R7 (including C4 and C5) were cloned and sequenced. Nucleotide sequence comparison showed that all 12 aptamers comprised the GUUG motif, which may represent the RNA binding site for EhCFIm25. The fact that this motif is also present in the 3′UTR of the *EhPgp5* gene that interacts with EhCFIm25^21,24^ prompted us to evaluate the relevance of this sequence in the aptamer-EhCFIm25 interaction. To this end, we performed a REMSA using the 3′UTR of two genes that carry the GUUG sequence (Fig. 2A). The *E*. *histolytica* genes coding for thioredoxin and 60S ribosomal protein L7 (accession number EHI_026340 and EHI_192110, respectively) were selected because RNA sequencing experiments had shown that they contain two functional alternative poly(A) sites^46^. The REMSA showed that the 3′UTR of both genes formed a complex with the endogenous protein contained in total protein extract from *E*. *histolytica*, as well as with the purified recombinant EhCFIm25 (Fig. 2B). To assess the relevance of the GUUG motif, we designed an oligonucleotide with a random sequence that contains the GUUG motif. As expected, EhCFIm25 formed a complex with this GUUG probe, whereas replacement of the GUUG sequence with the CAAC motif (in a CAAC probe) totally abolished EhCFIm25 binding. Moreover, the GUUG-probe acted as a specific competitor in REMSAs using the 3′UTR of the EHI_026340 and EHI_192110 genes as probes (Fig. 2C–E).

### Protein and RNA modelling, molecular docking, and molecular dynamics simulations

**Figure 3:**
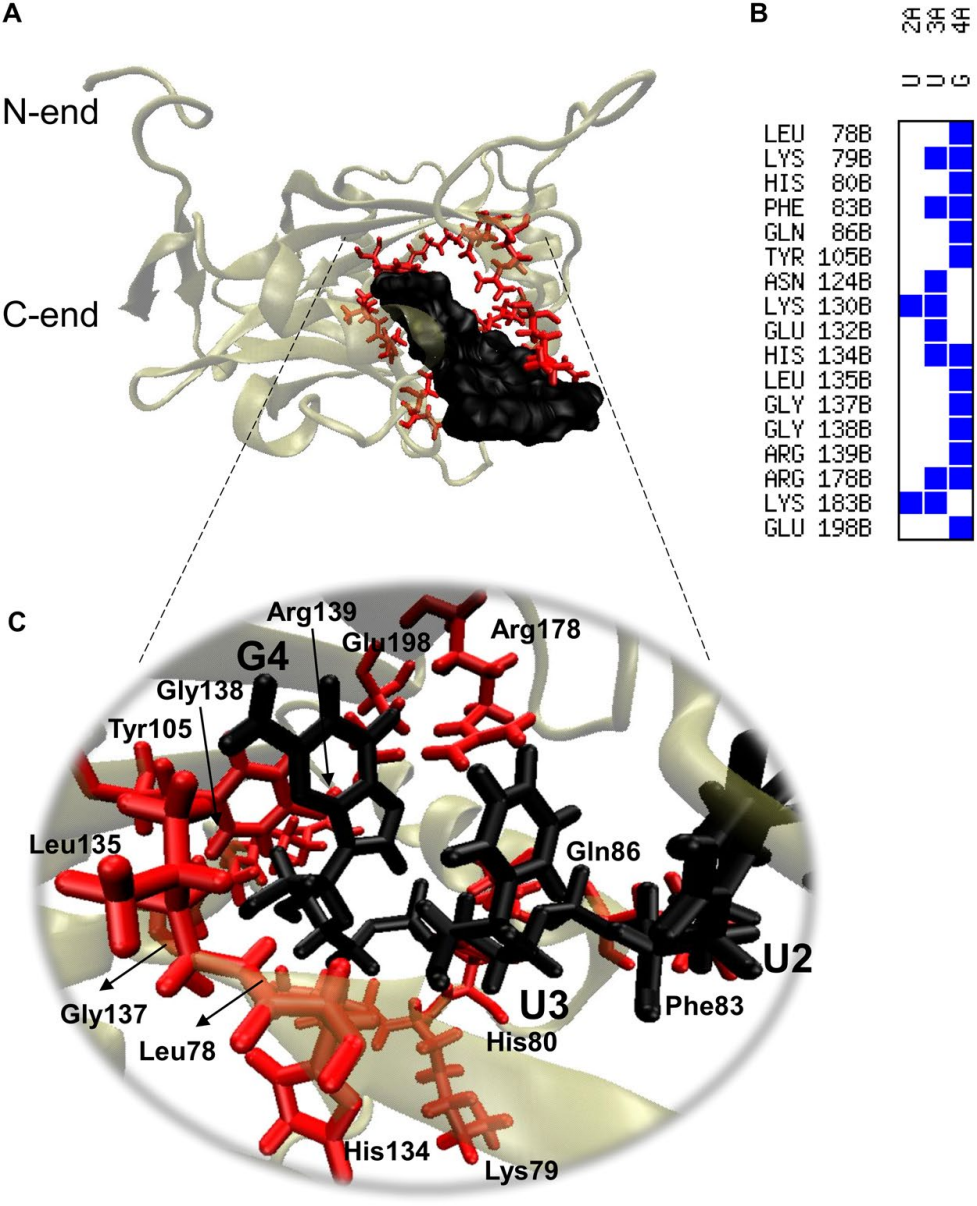
Interactions between EhCFIm25 and the GUUG motif. (**A**) Graphic representation of the average structure of EhCFIm25 complexed to the GUUG motif. (**B**) Contact map between the aa in EhCFIm25 and the nucleotides (U2, U3 and G4) in the GUUG motif. (**C**) A close-up view of the interaction zone between selected aa in EhCFIm25 (in red) and U2, U3 and G4 in the GUUG fragment (in black).

**Figure 4:**
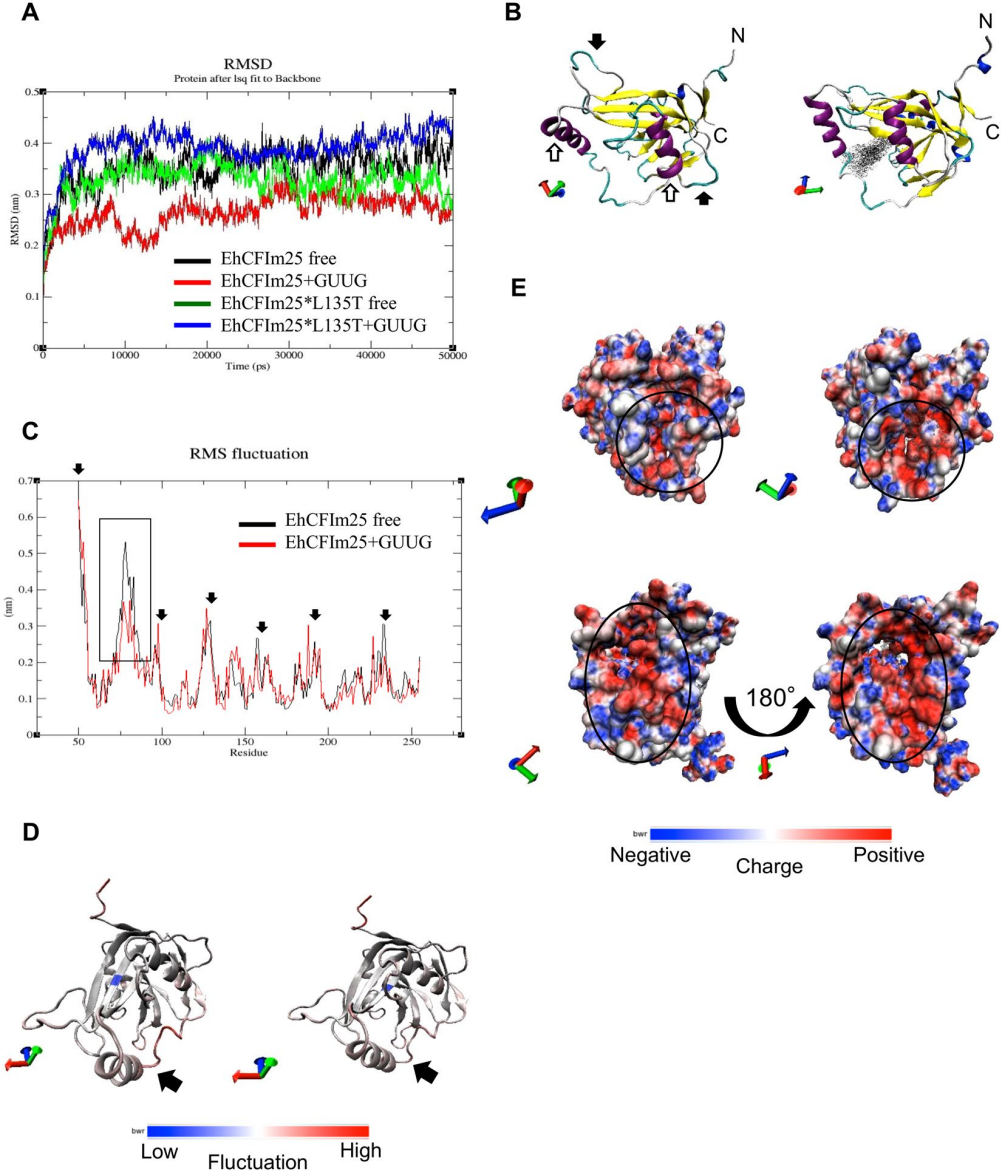
Molecular dynamics simulations of free and GUUG-binding EhCFIm25 proteins. (**A**) Change over time in root mean square deviation of Cα (RMSD). Data on the mutant EhCFIm25*L135T protein were included as control. (**B**) Average structure of the free (left) and GUUG-binding (right) proteins. Black arrow: unstable loop; white arrow: disordered alpha helix. (**C**) Root mean square fluctuations of Cα coordinates (RMSF). Arrows show the most flexible regions in both systems. The box indicates the region (70–90 aa) that differs in flexibility when comparing the two systems. (**D**) Fluctuations of aa in the average structure of free (left) and GUUG-binding (right) protein. Black arrows indicate the 70–90 aa region with the greatest fluctuation. (**E**) The surface electrostatic potential of the free (left) and GUUG-binding (right) proteins. Upper panel: front view; lower panel: rear view. The circle shows the RNA-interacting area.

To gain insight into how the EhCFIm25 protein binds to the GUUG motif, we compared data generated in MD simulations of EhCFIm25 alone or bound to the GUUG motif. We used the three-dimensional structure of the predicted RNA-protein complex with the best score in a docking assay. Results of MD simulations showed that the EhCFIm25-GUUG interaction mainly occurs through the nitrogenous base guanine 4 in the RNA, and some positively charged aa (Lys, Arg and His), some aromatic aa (Phe and Tyr), and the Leu135 residue previously known to be important for the RNA binding activity of EhCFIm25 (Fig. 3)^24^. We also compared the changes over time (during 50 ns) in the structure of free *vs*. RNA-bound EhCFIm25 at 300 ºK. Under both experimental conditions, the root mean square deviation (RMSD) reached equilibrium after 15 ns of simulation (Fig. 4A). The free protein presented fluctuations in the 0.3–0.4 nm range, while the GUUG-interacting protein fluctuated less (0.2–0.3 nm) - suggesting that the interaction with RNA stabilized the protein’s structure as a whole. In contrast, the GUUG motif had a destabilizing effect on the mutant EhCFIm25*L135T protein, which confirms the poor RNA binding capacity observed *in vitro*^24^. Consistently, analysis of the trajectories in the MD simulations of the free and GUUG-bound proteins confirmed that EhCFIm25 is stabilized in the presence of the RNA molecule (Supplementary videos S1 and S2). Indeed, the average structure in the last 30 ns of the simulation showed that the GUUG-bound protein is more compact than the free EhCFIm25; the latter has a pair of unstable loops and disordered alpha helices (Fig. 4B).

To establish how the interaction with RNA affects the behaviour of the protein’s aa, we compared the root mean square fluctuation (RMSF) of EhCFIm25 in the presence or absence of the GUUG fragment during the last 30 ns of the MD simulation (Fig. 4C). Under both conditions, the structure contained the same flexible regions (indicated by black filled arrows in the Figure). However, the GUUG-interacting protein displayed lower RMSF values in the 70–90 aa region (box). Examination of EhCFIm25’s three-dimensional structure using the VMD software confirmed that the amino-terminal region and the 70–90 aa region (corresponding to an alpha helix and a loop) fluctuated the most, whereas the rest of the structure was more stable in both the bound and free proteins (Fig. 4D). In line with the RMSF data, the 70–90 aa region appeared to be stabilized by structure gain (transition from a loop to an alpha helix) in the GUUG-interacting EhCFIm25; this suggests that the 70–90 aa region is closely involved in RNA binding (Supplementary videos S1 and S2). Lastly, we calculated the electrostatic potential of EhCFIm25’s surface. As shown in Fig. 4E, the RNA binding site (circle) is more electropositive in the GUUG-interacting protein than in the free protein - suggesting that electrostatic interactions are involved in the formation of the GUUG-EhCFIm25 complex.

### C4 and C5 aptamers inhibit proliferation and induce the death of *E*. *histolytica* trophozoites

**Figure 5:**
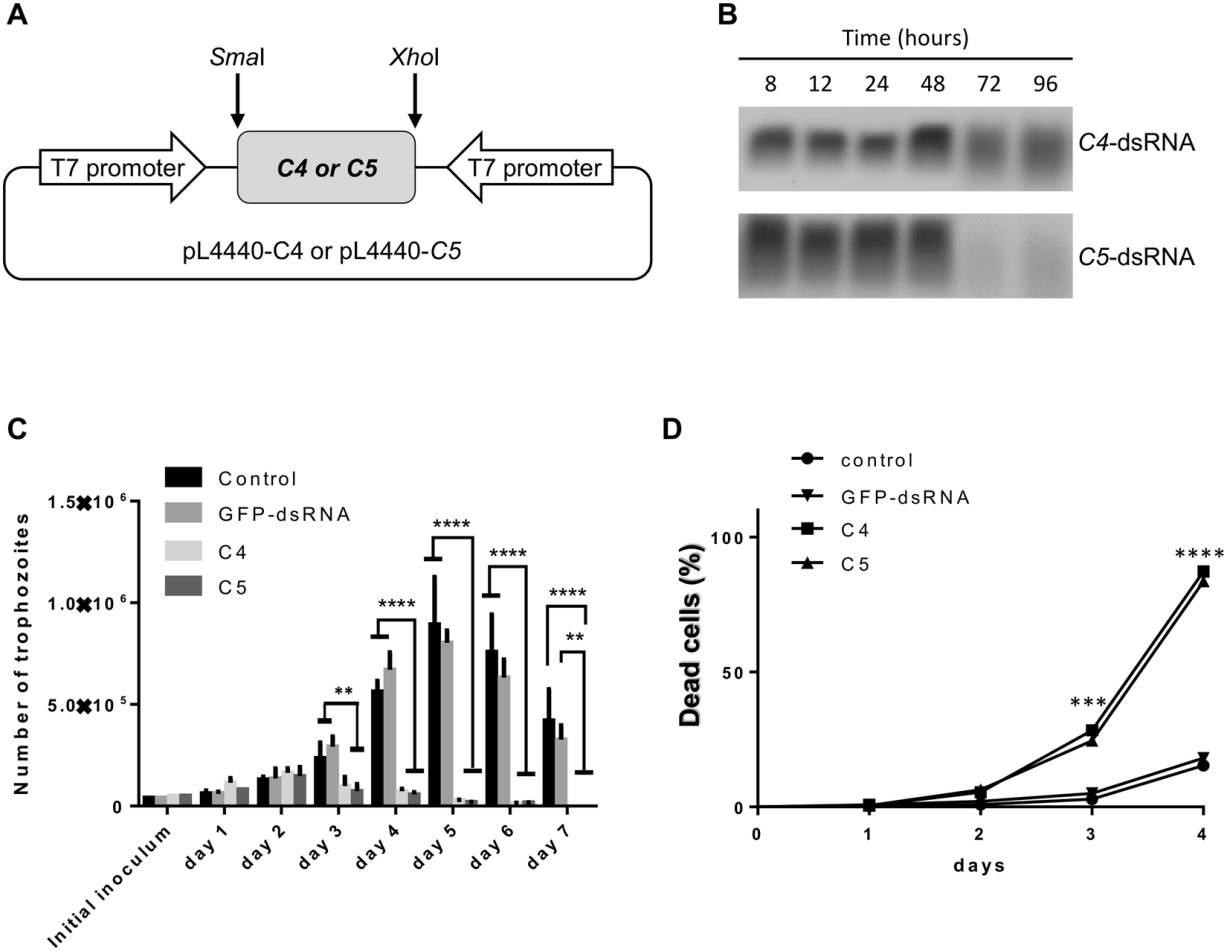
Effect of C4 and C5 aptamers on the proliferation and viability of *E*. *histolytica* trophozoites. (**A**) pL4440-C4/C5 plasmid constructs. (**B**) Detection of dsRNA in TYI-S-33 medium. (**C**–**D**) Trophozoites were treated with *C4*- or *C5*-dsRNA (100 µl/ml) and incubated at 37 °C. Each day, the cell count was determined (**C**), and cell viability was assessed in a Trypan blue assay (**D**). Trophozoites grown in the absence of dsRNA (control) or in the presence of *gfp*-dsRNA were also used. Data were analysed in a two-way analysis of variance or a T-test, as appropriate. **p < 0.01, ***p < 0.001 and ****p < 0.0001.

It has been previously reported that some vectors can allow the expression or delivery of aptamers inside cells, where they can reach their nuclear or cytoplasmic targets^47–49^. To deliver C4 and C5 aptamers into *E*. *histolytica*, we took advantage of our previous observation whereby soaking trophozoites in medium with dsRNA produced in bacteria results in uptake^29,45,50^. We cloned the cDNA sequences corresponding to the C4 or C5 aptamers into a pL4440 plasmid (Fig. 5A), expressed the aptamers in bacteria, and performed parasite soaking experiments with the resulting *C4-* and *C5*-dsRNA. Both *C4*- and *C5*-dsRNA were identified by electrophoresis in the culture medium on day four of the experiment - indicating that dsRNA was available for ingestion by trophozoites (Fig. 5B). Growth curves showed that both C4 and C5 aptamers significantly reduced cell proliferation, relative to control experiments. For example, proliferation at 96 h was reduced by ~80% in the presence of aptamers (Fig. 5C). Consistently, cultures exposed to *C4*- or *C5*-dsRNA contained a higher number of dead trophozoites (~80%) than control groups did (~20%) (Fig. 5D). We did not observe any significant differences between trophozoites grown in standard conditions or with *gfp*-dsRNA; hence, ingestion of unrelated dsRNA or random contaminant *E*. *coli* RNA are not responsible for growth inhibition. This observation strengthens the relationship between the C4 and C5 aptamers and the observed phenotype. BLAST searches in *E*. *histolytica* genome, including genes encoding known and unknown proteins, showed that neither the selected strand nor the complementary strand of C4 and C5 dsDNA displayed homology with any of the parasite’s gene; so it is unlikely that C4 and C5 aptamers could act via RNAi and affect the expression of any parasite proteins.

## Discussion

In the present study, we developed RNA aptamers that targeted the EhCFIm25 protein in *E*. *histolytica* and reduced trophozoite survival. To the best of our knowledge, this is the first study to have applied aptamer-based approaches in *E*. *histolytica* and the second to have isolated aptamers that can distinguish between two proteins that differ only with regard to a single point mutation. By using a new contrast screening strategy with SELEX (five cycles), Chen *et al*. (2005) recently isolated an RNA aptamer bound more strongly to a mutant p53 protein (R175H) than to the wild-type protein^51^. Our use of seven rounds of selection in a conventional and highly specific SELEX protocol enabled us to generate two aptamers (C4 and C5) that only recognize and bind the wild-type EhCFIm25, as confirmed by REMSAs with extracts from HeLa cells or *T*. *cruzi*, and the mutant protein that is still able to bind RNA^24^. Further experiments, such as ELONA and Slot-Blot, should be done to measure their affinity and specificity.

Since EhCFIm25 is an RNA-binding protein, its interaction with C4 and C5 depends on the RNAs’ sequence and the three-dimensional structure. Given that all the isolated aptamers and all the 3′UTRs linked by EhCFIm25 contain the GUUG sequence, we hypothesize that the latter is the canonical RNA motif recognized *in vitro* by EhCFIm25. *In silico* analysis of the EhCFIm25-GUUG complex revealed that the interaction takes place in an RNA binding pocket that is in the same position as in the crystal structure of the human CFIm25 protein complexed with the UGUAU motif^52^ - despite differences in the aa and nucleotide sequences (Supplementary Figure S3). On the basis of data obtained from the computational analysis of 32 crystallized RNA-protein complexes Jones *et al*. suggested that aromatic and positive charged aa have a fundamental role in RNA binding^53^. Moreover, the interaction with the GUUG sequence stabilizes EhCFIm25 - making it more compact, and structuring some regions of the protein. Notably, the 70–90 aa region that folds into an alpha helix and the loop seems to be involved in the positioning of the GUUG molecule in EhCFIm25′s RNA binding pocket. These structural transitions from loops to alpha helices are considered to be “molecular switches” for interaction with nucleic acids^54^. Furthermore, the formation of a cluster of positively charged aa may promote electrostatic interaction between EhCFIm25 and the GUUG fragment. With a view to the future clinical applications for C4 and C5 aptamers, it must be kept in mind that the GUUG motif bound by EhCFIm25 differs from the UGUA sequence recognized by the human homolog^19^. Although the human and parasite CFIm25 proteins have similar three-dimensional structures, there are marked differences between the respective aa sequences and proteins display sequence identity of only 32%^21^. Given that the C4 and C5 aptamers did not bind to human proteins (including CFIm25), they would be unlikely to affect the polyadenylation process in the hosts’ cells. As mentioned above, additional ongoing experiments should confirm the specificity, selectivity and affinity of the interaction between C4/C5 and EhCFIm25, as a first step towards the aptamers’ use as therapeutic tools.

Several researchers have reported that disrupting 3′UTR processing provides opportunities for the development of new anti-parasitic compounds^25–28^. In *E*. *histolytica*, we have observed that EhCFIm25 silencing alters poly(A) site selection and parasite survival^29^. Since EhCFIm25 controls the selection of poly(A) sites, EhCFIm25 sequestration is expected to modify the stability of the mRNAs produced. This may result in an overall alteration of gene expression and then rapid death of the parasite. Moreover, the presence of aptamers might also affect the availability and/or function of proteins that interact with EhCFIm25 (such as EhPAP^21^ and EhPC4) and proteins involved in other mRNA processing events (as has been described in humans)^55,56^. Consistently, our molecular protein-protein docking experiments indicated that EhCFIm25’s interaction with GUUG, EhPAP and EhPC4 involves distinct binding sites (Supplementary Figure S4). Hence, blocking of EhCFIm25 by C4 or C5 aptamers (leading to an increase in protein stability) may also have additional effects on EhCFIm25’s interaction with EhPAP and EhPC4 - both of which have fundamental roles in gene expression. We conclude that aptamers represent a powerful new biotechnological tool for blocking polyadenylation in *E*. *histolytica*.

Most conventional means of introducing nucleic acids to cells are based on lipofectamine transfection systems. In the present work, we observed that the dsRNA soaking method (originally used to silence gene expression in *E. histolytica*)^29,45,50^ represents an efficient, reproducible, fast, easy-to-implement strategy for blocking a nuclear target protein like EhCFIm25. In the literature, aptamers linked to siRNA (aptamer-siRNA chimeras) are used to deliver both molecules to cells^57^. We took advantage from *E*. *histolytica* properties to uptake dsRNA to deliver the aptamers C4 and C5 synthetized in bacteria under dsRNA form. Although the mechanism by which dsRNA enter into *E*. *histolytica* is not known, several proteins sharing similarities with eukaryotic RNAi factors have been described, such as an RNaseIII-like protein, as well as orthologues of RdRP and Argonaute^58,59^, among others. So, we expected that dsRNA (aptamer-anti/aptamer construct) could be converted to ssRNA molecules intracellularly as it is done during the RNAi phenomenon. A single inoculation of C4 or C5 aptamer had a huge inhibitory effect on the proliferation and viability of *E*. *histolytica* trophozoites. Since aptamer sequences have no homology with any known DNA sequence within the amoeba genome, we assumed that ssRNA aptamers bind the endogenous EhCFIm25 *in vivo* as they do *in vitro*.

In conclusion, we developed RNA aptamers that contain the GUUG motif (recognized by the EhCFIm25 factor) and that effectively control *E*. *histolytica*’s survival *in vitro*. These valuable findings suggest that aptamers could be used to control *E*. *histolytica* during the development of amoebiasis or to eradicate residual trophozoites during antibiotic treatment. In the context of parasitic infections, several other groups have developed aptamers that specifically identify *T*. *cruzi* and *Plasmodium* in blood, and *Leishmania* in the sand fly vector^60–65^. Other researchers have used aptamers to assess drug efficacy in Chagas disease^66^. In *Leishmania*, the SELEX process has been used to generate aptamers that bind to nuclear proteins such as histone H2A^67–69^ and the poly(A)-binding protein^70^; however, the researchers did not evaluate the aptamers’ effect on parasite survival. Future investigations will focus on clinical strategies for aptamer delivery against human pathogens *in vivo*. The results of the present proof-of-concept study in *E*. *histolytica* may promote the use of aptamers to treat globally challenging parasitic diseases.

## Electronic supplementary material

Supplementary video S1.A molecular dynamics simulation of free EhCFIm25 protein.

Supplementary video S2. A molecular dynamics simulation of EhCFIm25 interacting with the GUUG fragment.

Supplementary figures S1 to S4
