# Supplementary material for: Targeting the polyadenylation factor EhCFIm25 with RNA aptamers controls survival in Entamoeba histolytica
Source: Sci Rep. 2018 Apr 9;8:5720. doi: 10.1038/s41598-018-23997-w (PMC5890266; doi:10.1038/s41598-018-23997-w)
Supplement: Supplementary file 3 — Supplementary figures S1 to S4 [file 41598_2018_23997_MOESM3_ESM.pdf]

Targeting the polyadenylation factor EhCFIm25 with RNA aptamers controls survival in *Entamoeba histolytica*

Juan David Ospina-Villa, Alexandre Dufour, Christian Weber, Esther Ramirez-Moreno, Absalom Zamorano-Carrillo, Nancy Guillen, César Lopez-Camarillo, Laurence A Marchat

Supplementary Figure S1

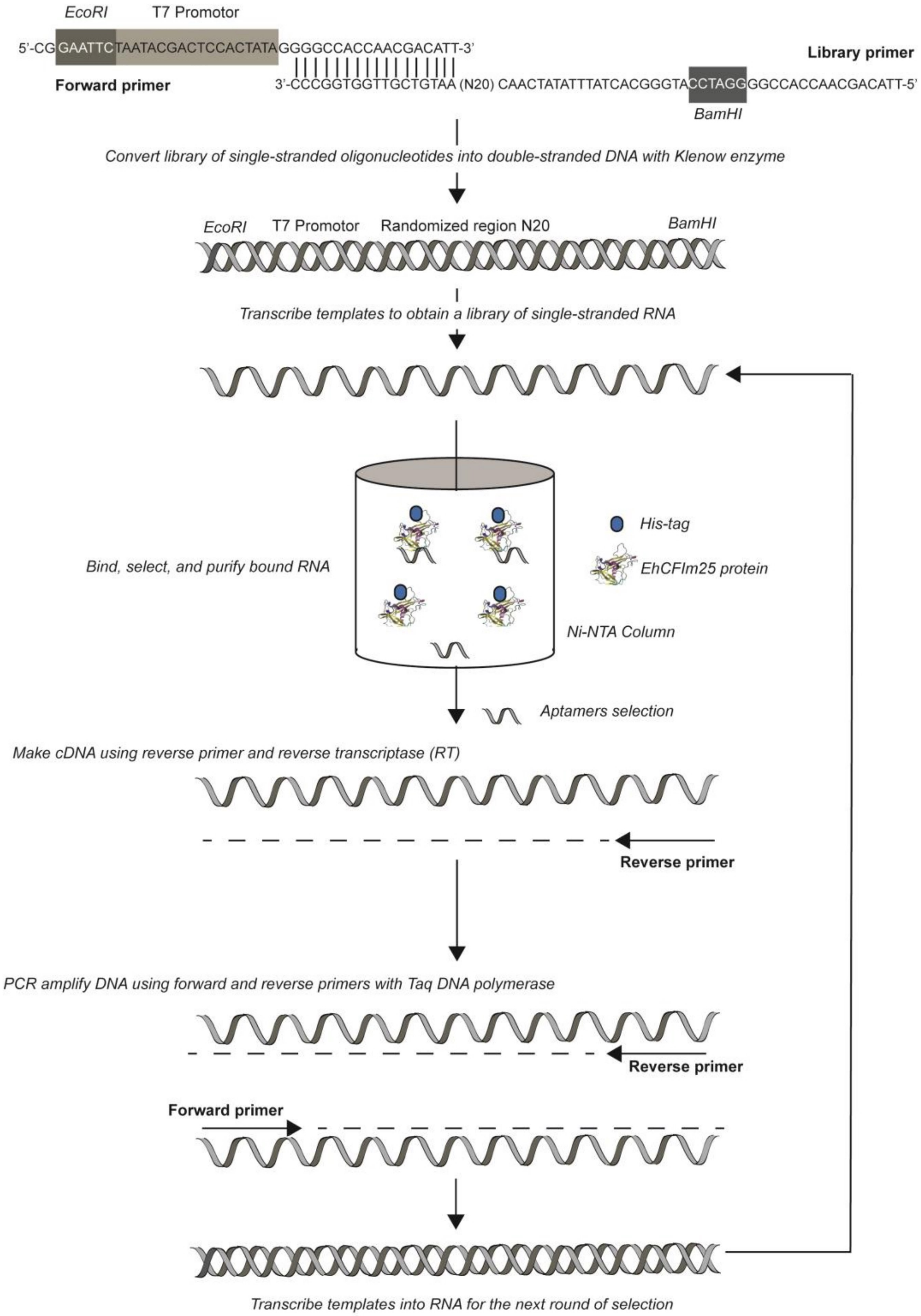

Supplementary Figure S1. Schematic representation of the SELEX strategy. The first step involves the conversion of a single-stranded (ss) oligonucleotides library into double-stranded (ds) DNA with the Klenow enzyme. Then, the dsDNA library is *in vitro* transcribed into RNA that interacts with the recombinant EhCFIm25 protein immobilized on a Ni-NTA column. ssRNA that do not interact with the protein are discarded, while interacting ssRNA are amplified by RT-PCR and *in vitro* transcribed to generate a new ssRNA library for subsequent rounds of selection.

Supplementary Figure S2

Nucleotide sequence of aptamer C4:

5'-
GGGCCACCAACGACAUUGAUCUUAUUUCAUACCGGGAGUUGAUAUAAAUA
GUGCCCAUGGAUCCCC-3‘

Nucleotide sequence of aptamer C5:

5'-
GGGCCACCAACGACAUUCCUGCGUGCCAGAGCACAUUGUUGAUAUAAAUA
GUGCCCAUGGAUCCCC-3'

Supplementary Figure S2. Nucleotide sequences of aptamers C4 and C5.

Supplementary Figure S3

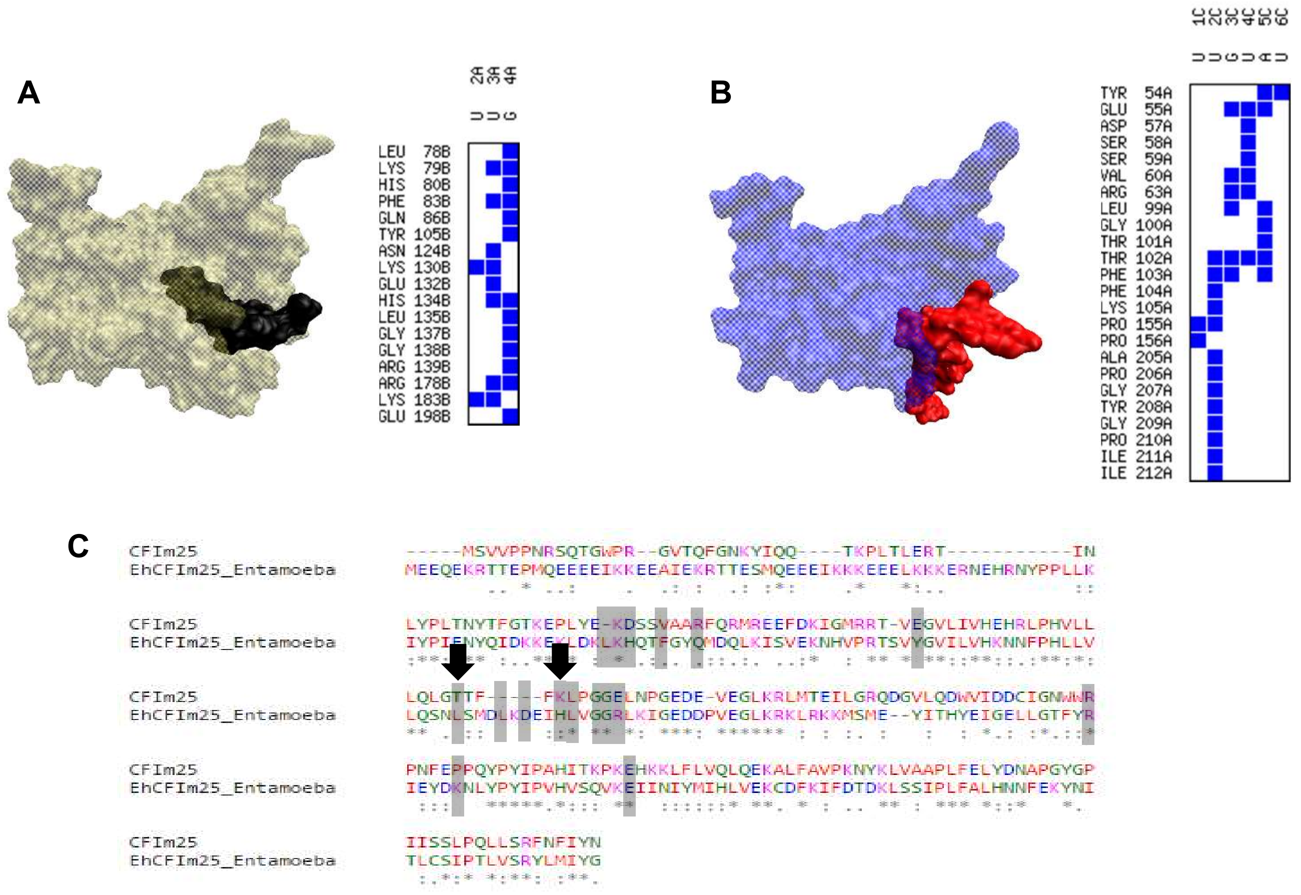

Supplementary Figure S3. Comparison between amoeba and human RNA-CFIm25 complexes. Comparison between the three-dimensional structures of EhCFIm25+GUUG complex obtained by Docking (A) and the crystal structure of the CFIm25 protein interacting with the UGUAUU Motif (B). Contact maps are at the right in each panel. C) Alignment of amino acid sequences of EhCFIm25 and human CFIm25 proteins. Black shadows, position of amino acids of EhCFIm25 that are in contact with the GUUG motif; black arrows, amino acids of both proteins that interact with RNA

Supplementary Figure S4

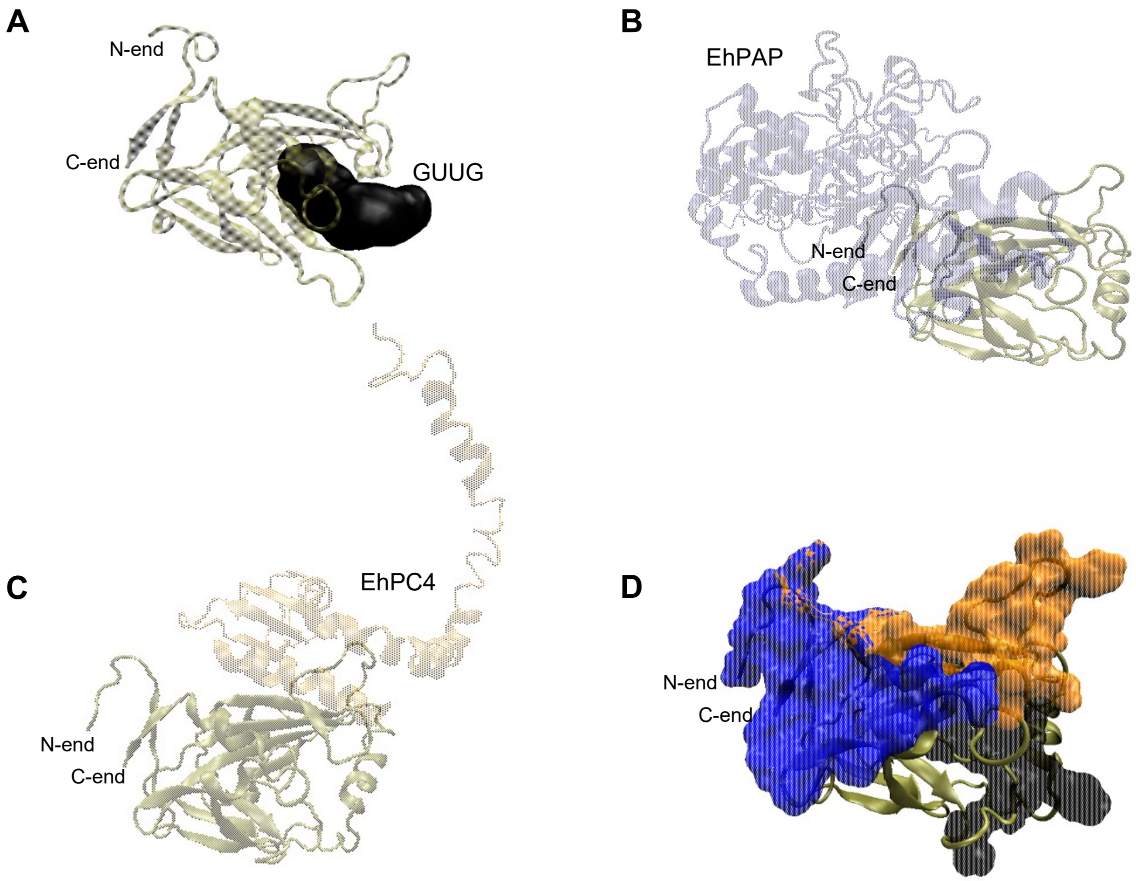

Supplementary Figure S4. Interactions domains in EhCFIm25. A-C) Molecular Docking to predict interaction sites of the EhCFIm25 protein (tan shadow) with the GUUG fragment (black bead) (A), EhPAP (blue shadow) (B) and EhPC4 (orange shadow) (C). D) Graphical representation of EhCFIm25 (tan) interacting withthe GUUG sequence (black surface), EhPAP (blue surface), and EhPC4 (orange surface).
